# Giant Magnetoresistive Biosensors for Time-Domain Magnetorelaxometry: A Theoretical Investigation and Progress Toward an Immunoassay

**DOI:** 10.1038/srep45493

**Published:** 2017-04-04

**Authors:** Chih-Cheng Huang, Xiahan Zhou, Drew A. Hall

**Affiliations:** 1 University of California – San Diego, Materials Science and Engineering Program, La Jolla, CA 92093, USA; 2 University of California – San Diego, Department of Electrical and Computer Engineering, La Jolla, CA 92093, USA

## Abstract

Magnetorelaxometry (MRX) is a promising new biosensing technique for point-of-care diagnostics. Historically, magnetic sensors have been primarily used to monitor the stray field of magnetic nanoparticles bound to analytes of interest for immunoassays and flow cytometers. In MRX, the magnetic nanoparticles (MNPs) are first magnetized and then the temporal response is monitored after removing the magnetic field. This new sensing modality is insensitive to the magnetic field homogeneity making it more amenable to low-power portable applications. In this work, we systematically investigated time-domain MRX by measuring the signal dependence on the applied field, magnetization time, and magnetic core size. The extracted characteristic times varied for different magnetic MNPs, exhibiting unique magnetic signatures. We also measured the signal contribution based on the MNP location and correlated the coverage with measured signal amplitude. Lastly, we demonstrated, for the first time, a GMR-based time-domain MRX bioassay. This approach validates the feasibility of immunoassays using GMR-based MRX and provides an alternative platform for point-of-care diagnostics.

Dramatic improvements in medicine and the healthcare system over the past century have increased the average life expectancy worldwide as a result of better understanding of disease processes, new treatments, and advances in pharmaceuticals^1^. Yet, the largest contributor to this advancement is the earlier diagnoses of life threatening diseases, where treatments are much more effective and physicians have more options. Historically such medical decision making was primarily symptom driven whereas today it is increasingly reliant on molecular tests that analyze patient samples for disease-specific biomarkers (e.g., proteins, DNA, etc.). Current clinical biomarker detection technologies, such as colorimetric and fluorescent enzyme-linked-immunosorbent assays (ELISA), have proven to be effective, yet are confined to centralized laboratories, have time consuming and labor intensive protocols, and require expensive equipment^2^,^3^. These limitations have fueled research into alternative biosensing technologies that require less sample preparation and are more portable.

For the past two decades, magnetic biosensors have received considerable attention as they offer several key advantages over conventional and competing sensing methods^4^,^5^,^6^,^7^,^8^,^9^,^10^,^11^,^12^,^13^,^14^,^15^,^16^,^17^,^18^. Like an ELISA, a magnetic immunoassay (MIA) relies on two antibodies that form a sandwich structure around the biomarker of interest to achieve high specificity. However, the optical label in an ELISA is replaced with a 10–50 nm magnetic nanoparticle (MNP) in the MIA. This switch of label has been shown to retain sensitivity in unprocessed samples due to the lack of magnetic background signal^5^, reduce the need for tedious sample preparation^19^, allow for sample manipulation with magnetic fields^20^,^21^, and enable real-time monitoring of the binding kinetics^4^. However, the minute signal from the MNPs requires specialized magnetic sensors to be detected.

Early work developing magnetic biosensors can be traced back to 1990’s, and, since then, researchers have demonstrated biosensing using a host of different magnetic sensors including superconducting quantum interference devices (SQUIDs)^22^, inductive sensors^23^, hall effect sensors^20^,^21^, flux-gate magnetometers^24^, and magnetoresistive (MR)-based sensors^15^,^25^,^26^,^27^. Among the many magnetic sensors available today, MR-based devices standout for point-of-care (POC) applications. In addition to the inherent advantages of magnetic biosensing, MR biosensors can be operated at room temperature, have high low-field sensitivity, and have comparably high transduction efficiency. These MR-based sensors operate on a quantum mechanical effect (either spin-dependent scattering or tunneling) where the resistance is proportional to the magnetic field with magnetoresistance ratios ranging from 5% to >200% for modern devices^8^,^28^.

Previously, these MR biosensors utilized static magnetometry where one detects the MNP’s stray field in response to a DC or fixed frequency AC magnetic field. However, this technique requires a homogeneous magnetic field, complex readout electronics, and substantial signal processing to extract the minute signal of interest, all of which are challenging to do in a power constrained, remote POC environment. An alternative approach is based on magnetorelaxometry (MRX) where one detects the relaxation signature in response to a pulsed magnetic field. This technique removes the need for a homogenous magnetic field and requires comparably simpler readout electronics and signal processing. Figure 1 illustrates how this technique is applied for magnetic biosensing. In the absence of an external magnetic field, the magnetic moments of the superparamagnetic MNPs tethered to the surface of the sensor are randomly oriented resulting in zero net field (Fig. 1a). Then, a magnetic field (*H*_A_) is applied that magnetizes and aligns all the MNPs. The stray field from the MNP opposes the applied field resulting in a small change in resistance in the underlying MR sensor (Fig. 1b). Note, this is the region of operation for static magnetometry. However, in MRX, the applied magnetic field is then switched off and the sensors are monitored as the MNPs gradually relax to their equilibrium state (Fig. 1c). This relaxation occurs due to Néel and Brownian relaxation. Néel relaxation is the result of internal magnetic domain movement within the MNP whereas Brownian relaxation is the rigid rotation of the entire MNP. Since the MNPs are tethered to the surface of the sensor via antibodies or other molecular recognition elements, the relaxation process is predominantly Néel based. This relaxation signal can be measured in either the frequency- or time-domain. The frequency-domain technique uses a continuous AC magnetic field to measure the in-phase and out-of-phase component of the susceptibility whereas the time-domain technique measures the temporal response due to a pulsed magnetic field. In a POC setting, the time-domain technique generally leads to a simpler implementation, but requires careful understanding of the factors that influence the signal, which, in the past, has limited the progress of this technique.

In this paper, we propose a novel time-domain MRX-based giant magnetoresistance (GMR) biosensor to observe Néel relaxation of tethered MNPs. To investigate this, we designed an ultrafast electromagnet with a switching time less than 5 μs (slew rate >1,000 T/s), which is much faster than the state-of-the-art with a 400 μs. switching time (slew rate of 37 T/s)^29^, to minimize the deadzone. Low-noise readout electronics were designed to capture the relaxation signal. The effect of the applied magnetic field amplitude (*H*_*A*_) and magnetization time (*t*_*mag*_) were explored to understand their influence on the relaxation process. The results show excellent agreement with the empirical trend describing the relaxation based on natural-log behavior. We use these findings to optimize the system and perform a proof-of-principle magnetic immunoassay, which is, to the best of our knowledge, the first time that GMR sensors have been reported for an MRX bioassay.

## Results

The measurement setup consisted of an 8 × 10 GMR sensor array (MagArray Technologies, Inc.) placed inside an electromagnet (custom designed) connected to readout circuitry (custom designed) as shown in Fig. 2 (described in detail in the Methods section). Due to the correlated double sampling technique (described in the Methods section), no magnetic shielding was required. Each sensor contained multiple GMR stripes to increase the surface area while maintaining the high aspect ratio of each stripe needed to keep the sensing layer stable with a nominal resistance of 1.7 kΩ and a magnetoresistance ratio of 11.5% (Fig. 2c). Streptavidin-coated MNPs (SHS-30-01, Ocean NanoTechnologies) were drop-casted on the sensors in the presence of an alternating field (50 Oe at 200 Hz) and allowed to dry before the experimental investigation of characteristic time, coverage, and signal dependence (over applied field and magnetization time). Prior to drop-casting, select sensors were covered with epoxy to prevent the MNPs from being in close enough proximity to the sensor (~200 nm) to create a detectable signal. These epoxy-coated sensors were used as negative controls (reference sensors) while all others were active sensors with MNPs. For magnetic immunoassay experiments, the reference sensors were coated with Bovine serum albumin (BSA) while active sensors were functionalized with biotin, which facilitated binding with the MNPs through the high affinity streptavidin-biotin interaction. With the above setup and experimental procedures, we successfully monitored the relaxation process of MNPs. The active sensors exhibited a natural log-like response with a characteristic time of 3.3*t*_*mag*_ while the reference sensors showed no response. Next, we extended the investigation to extract the MNP coverage and confirmed it with scanning electron microscope (SEM) analysis. The measured signal was found to be proportional to the MNP concentration. To improve the empirical and theoretical study of time-domain MRX, we investigated the signal dependence on the applied field, magnetization time, and MNP size/composition. The results showed that other contributors (i.e. temperature and MNP dispersity) need to be considered as well. The measurements of different MNPs showed that each has a unique *t*_c_ roughly proportional to the core volume. Lastly, we performed a magnetic immunoassay to demonstrate the feasibility of this approach.

### Investigation of Characteristic Time

Once the MNP is magnetized and the field is removed, there is insufficient energy to keep the moment of the MNP fixed. There are two mechanisms by which this loss of energy, or relaxation process, can occur. With two competing processes, the relaxation time will depend on the faster of the two mechanisms. Néel relaxation follows an exponential decay relationship when the MNPs are monodisperse^30^,^31^,^32^,^33^, and depends on the core volume and anisotropy of the MNP as described in Equation (1)


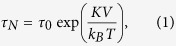


where *τ*_0_ is the attempt time (usually approximated as 10^−9^ sec), *K* is the anisotropy constant of the MNP, *V* is the core volume of the MNP, *k*_*B*_ is Boltzmann’s constant, and *T* is the absolute temperature. However, this relationship becomes natural log-like when considering particle-particle interactions and the size/shape distributions of the MNPs^30^,^31^,^32^,^33^,^34^,^35^,^36^. The time course magnetization during relaxation can be empirically described by the following equation


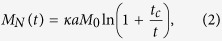


where *M*_*N*_ is the magnetization, *κ* is the surface coverage, *a* is a constant related to the magnetic viscosity, *M*_0_ is the initial MNP magnetization before the applied magnetic field is removed, *t* is the time after turning off the field, and *t*_c_ is the characteristic time that has a strong dependence on the applied magnetic field (*H*_*A*_) and magnetization time (*t*_*mag*_). It has been previously reported that *t*_*c*_ ≈ *t*_*mag*_ when the applied field is relatively small (*H*_*A*_ = 0.1 Oe)^31^. However, this has not been measured for more moderate magnetic fields that are appropriate for MR-based biosensing (20–100 Oe).

**Figure 1:**
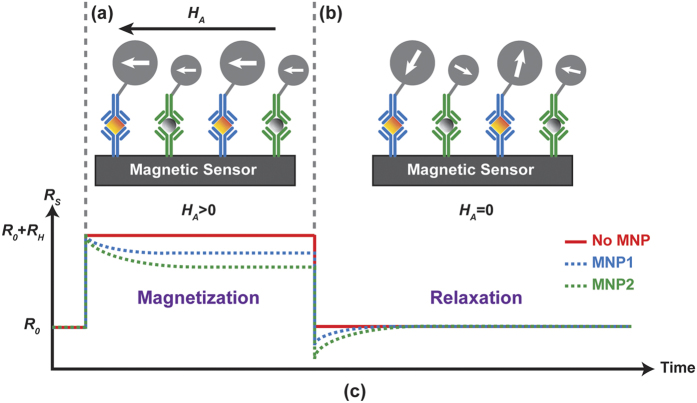
Illustration of time-domain magnetorelaxometry. (**a**) Magnetization phase (field *H*_*A*_ > 0 Oe) where the MNP magnetic moments are aligned to the applied field. (**b**) Relaxation phase (field **H**_**A**_ = 0 Oe) where the MNP magnetic moments gradually randomize. (**c**) The corresponding resistance of an MR sensor in response to the external magnetic field with and without MNPs.

**Figure 2:**
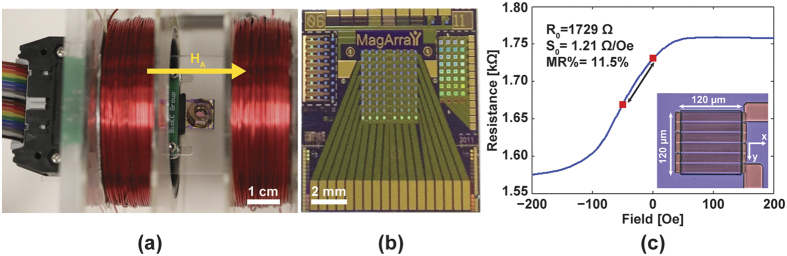
(**a**) Simplified measurement setup with electromagnet and sensor array. (**b**) Photograph of GMR sensor array. (**c**) Optical microscope image of a GMR sensor and measured magnetoresistance curve.

To measure the relaxation response, we restricted the particles to Néel relaxation by drop-casting a fixed volume of MNPs on the surface of the MR sensors and allowing them to dry while applying a magnetic field (50 Oe at 200 Hz). In such a configuration, the particles are rigidly attached and cannot undergo Brownian relaxation, thereby serving as a model system for the MIA. Moreover, the MNPs preferentially migrate into the trenches (the area between the stripes of GMR) in the presence of external field during the drying process, which significantly enhances the sensor response when MNPs are located close to the edge of trenches^37^,^38^. In these experiments, we followed the time-domain MRX procedure previously described (Fig. 1). The applied magnetic field (*H*_*A*_) was set to 50 Oe and pulsed for a duration of 100 ms. The field was subsequently collapsed in under 10 μs. The resulting resistances of the MR sensors were measured by applying a constant voltage across the sensors and integrating the current (described in the Methods section). The integrators were synchronized to start integrating after the electromagnet was turned off. The sensor array contained both active sensors (n = 29) and reference sensors (n = 20), which were coated with a thick epoxy to prevent the MNPs from being near the MR sensor thus quenching the relaxation signal.

**Figure 3:**
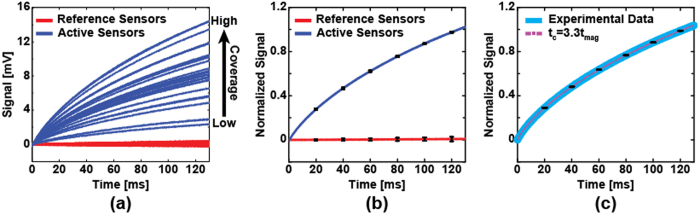
(**a**) Measured MRX signals from active sensors (blue, n = 29) and reference sensors (red, n = 20). The different amplitudes indicate non-uniform MNP coverage. (**b**) Normalized relaxation signals demonstrating the homogeneous relaxation behavior. The curves are the mean signal of reference sensors and active sensors, respectively. Error bars are ±1σ. (**c**) Comparison between experimental data and simulation exhibits good consistency of the characteristic time of 3.3*t*_*mag*_. Error bars are ±1σ.

The reference sensors all showed near zero signal, as expected, whereas the active sensors all exhibited a characteristic MRX signal with an amplitude ranging from 2 mV to 15 mV (Fig. 3a) due to the uncontrolled coverage on each sensor. When normalized by scaling the amplitudes to be the same (Fig. 3b), it is readily apparent that each sensor is measuring the same process, just scaled by the surface coverage. From the fitted data, we found that *t*_*c*_ = 3.3*t*_*mag*_ = 330 ms for *H*_*A*_ = 50 Oe and *t*_*mag*_ = 100 ms (Fig. 3c). Using magnetic modeling (described in the Methods section) to simulate the response of this system with the same *t*_*c*_, we found that the simulation results are in good agreement with the measured data. As will be shown later, *t*_*c*_ can be used as a unique magnetic signature for each type of MNP.

### Coverage Extraction

**Figure 4:**
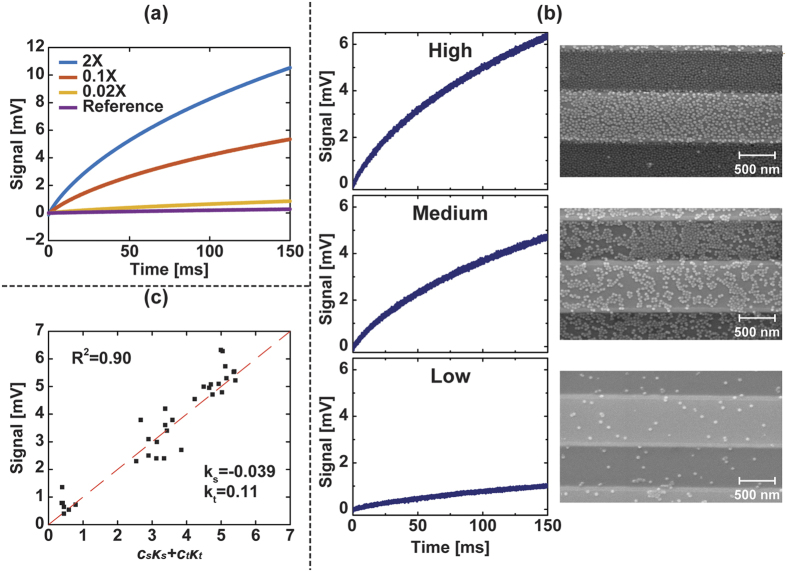
(**a**) Average signal under different MNP concentrations. (**b**) Measured relaxation signals and the corresponding SEM images. The three representative sensors, varied from low to high coverage of MNPs, exhibited high signal dependence over coverage. (**c**) Extraction of surface coverage showed different signal responses between stripe coverage and trench coverage. The fitted coefficients ***C***_***s***_ and ***C***_***t***_ are −0.039 and 0.11, respectively.

After verifying the natural log behavior of the relaxation signal and measuring the corresponding characteristic time, we investigated the signal dependence on the MNP coverage. For the detection of biomolecules labeled by MNPs, extraction of the MNP coverage is required to quantitatively retrieve the concentration of analytes and further deduce the ligand-receptor interaction characteristics, such as binding affinity and kinetics. In this section, we conducted the proof-of-principle experiments, extracting the coverage of MNPs in the absence of probe molecules (i.e., surface immobilization). For a single MNP, the signal would be highly dependent on the location within the sensor^38^. However, with moderate surface coverage, the signal per MNP is roughly constant and independent of location other than if it is on top of the sensor or in the trench next to the sensor^39^,^40^. Previously we were only able to calculate the relative MNP coverage based on the signal amplitude. To precisely extract the coverage parameter *κ* in Equation (2), we applied different MNP concentrations and imaged the sensors using a SEM after MRX measurements (Fig. 4). The number of MNPs on the sensor was calculated from the SEM images and compared with the corresponding measured MRX curve. The signal is dependent on the concentration of MNPs as shown in Fig. 4a, and the average signal at *t* = 150 ms is 0.86 mV, 5.34 mV, and 10.51 mV for 0.02×, 0.1×, and 2× the concentration of SHS-30 MNPs, respectively. It is important to note that the ratio of average signals between 0.02× and 0.1× the concentration (*Signal*_0.02×_/*Signal*_0.1×_ = 0.16) is similar to the ratio of their concentrations (*Conc*_0.02×_/*Conc*_0.1×_ = 0.2); however, this linear concentration dependence did not adequately represent the signal with higher MNP concentration (2×), since multi-layer MNP structures were formed at this high of concentration. Furthermore, high MNP concentration is not a realistic scenario for bioassays where a monolayer is the theoretical limit due to the surface ligand binding. Consequently, we focused on the 0.02× and 0.1× MNP concentrations to extract the coverage where the signal per particle is linear and the coverage is still monolayer (Fig. 4b). Since the design of the sensor geometry results in different signals that depend on the MNP position (i.e., on the stripe vs. trench)^37^, the total coverage over sensor area is not sufficient to address and extract the information of MNP coverage. Instead, the coverage should be evaluated over the GMR stripes and trenches (area between adjacent stripes), respectively. Equation (2) is modified accordingly to account for this dependency


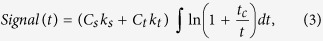


where *C*_*s*_ and *C*_*t*_ are dimensionless coefficients containing the magnetic viscosity and signal per particle on the sensor and trench, respectively, and *k*_*s*_ and *k*_*t*_ are the stripe and trench coverage in terms of percentage. From the measured data, we found that *C*_*s*_ = −0.039 ± 0.02 and *C*_*t*_ = 0.11 ± 0.018 (Fig. 4c). The inequality of *C*_*s*_ and *C*_*t*_ proves the positional dependence and reaffirms the previously reported result that the MNPs in the trenches contributed to signal more than the MNPs on the stripes^38^. The correlation between signal and extracted coverage coefficients exhibited strong consistency (*R*^2^ = 0.90) as shown in Fig. 4c.

### Signal Dependence on External Field and Magnetization Time

**Figure 5:**
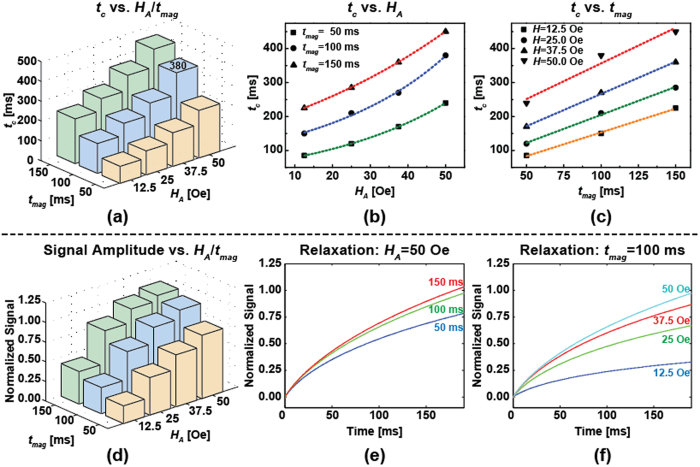
Measurement results showing: the effect of (**a**) **H**_**A**_ and **t**_**mag**_ on **t**_**c**_, (**b**) *t_c_* on *H_A_*, (**c**) *t_c_* on *t_mag_*, and (**d**) *H_A_* and *t_mag_* on the (normalized) signal amplitude as well as the relaxation signal under (**e**) *H_A_* = 50 Oe with increasing tmag and (**f**) under tmag = 100 ms with increasing *H_A_*.

Subsequently, we extended the experiment to measure the signal dependence on *H*_A_ and *t*_*mag*_ to optimize the time-domain MRX response. As expected, *t*_*c*_ has strong dependence on *H*_*A*_ and *t*_*mag*_ (Fig. 5a), varying from 85 ms to 450 ms in the given range of *H*_*A*_ and *t*_*mag*_. Based on our results, *t*_*c*_ has a quasi-linear relationship with *t*_*mag*_, while being exponentially dependent on *H*_*A*_ (Fig. 5b,c). The underlying theory still needs to be investigated to validate this observation. It should be noted that the extracted *t*_*c*_ = 380 ms at *H*_*A*_ = 50 Oe and *t*_*mag*_ = 100 ms is not the same as previously measured (*t*_*c*_ = 330 ms). We believe that this discrepancy is a result of different measurement temperatures (particularly here where the electromagnet was running for an extended duration resulting in an elevated temperature). Nevertheless, the signal amplitude followed the trend of *t*_*c*_, as expected, when sweeping *H*_*A*_ and *t*_*mag*_ (Fig. 5a). The normalized data, which were processed to remove the coverage effect, showed a positive correlation with *H*_*A*_ and *t*_*mag*_ (Fig. 5b,c). In terms of signal amplitude (Fig. 5d–f), the normalized signals show diminishing returns when increasing *H*_*A*_ and *t*_*mag*_, i.e. the increasing trend of the signal is not as obvious as *t*_*c*_ with increasing *H*_*A*_ and *t*_*mag*_.

### Characteristics of Time-domain MRX

**Figure 6:**
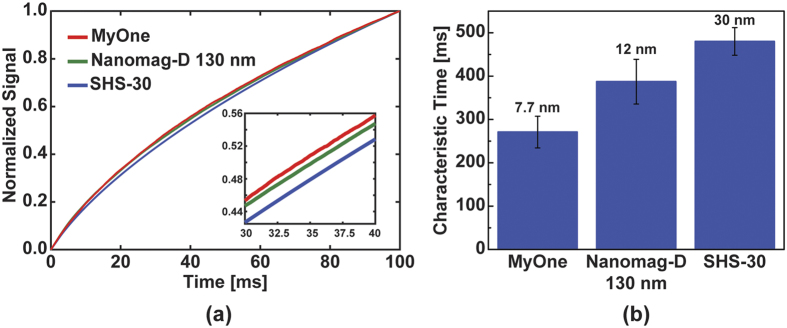
(**a**) Measured relaxation curves for different MNP and (**b**) the extracted characteristic time annotated with the core size of the MNP. Measurements repeated on multiple sensors (n = 62, 45, 49, respectively.) Error bars are ±1σ.

Based on the previous sections, we can enhance the signal through increasing the external field, magnetization time, and MNP concentration. In this experiment, we fixed *H*_*A*_ = 75 Oe and *t*_*mag*_ = 150 ms and investigated the signal from different MNPs. According to the literature and datasheets^41^,^42^,^43^, the mean cores size are 7.7 nm, 12 nm, and 30 nm for MyOne, Nanomag-D, and SHS-30, respectively. The normalized signals, which eliminate the effect of coverage, exhibited characteristic signatures unique to each MNP (Fig. 6a), and the extracted characteristic times of these three MNPs varied from 270 ms to 480 ms (Fig. 6b). The measurements were conducted under the same ambient temperature, hence the differences in the normalized signal must be from the different characteristic times. These results agree with the increased Néel relaxation time as described in Equation (1) where the time is dependent on the core volume. While time-domain MRX was substantially limited by the deadzone time (switching time) of the magnetic field in earlier work^31^,^33^, we have dramatically improved the time-domain MRX system with GMR sensors and successfully detected three kinds of MNPs.

### Progress Toward Magnetic Immunoassay

**Figure 7:**
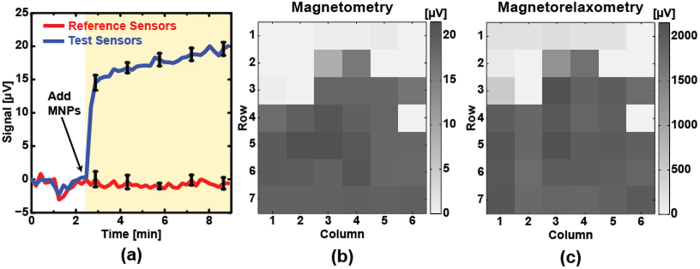
(**a**) Measured real-time magnetic immunoassay based on magnetometry, the curves are the mean signals of reference sensors (red, n = 8) and active sensors (blue, n = 25), respectively. Error bars represent ±1**σ**. Corresponding coverage map for (**b**) magnetometry and (**c**) magnetorelaxometry.

To demonstrate MRX as a biosensing technique, we performed an immunoassay and compared the results to the conventional magnetometry approach. In this experiment, the active sensors were functionalized with biotin whereas reference sensors were functionalized with BSA. The protocol used to functionalize the sensors is described in the Methods section. It should be noted that this protocol was designed specifically for the SHS-30 MNPs that have a zeta potential between −40 mV to −20 mV^44^,^45^. To compare both techniques, MRX measurements were taken before and after adding the streptavidin-coated MNPs to the assay. The assay was monitored in real-time using conventional magnetometry^6^ (Fig. 7a). As expected, the streptavidin-conjugated MNPs bound to the biotin on the surface of the active sensors. The reference sensors showed no signal, indicating no specific binding. The corresponding coverage maps are shown in Fig. 7b,c for magnetometry and magnetorelaxometry, respectively. Both coverage maps show a high degree of similarity, confirming the validity of the proposed technique. Since the noise is uncorrelated in the MRX measurements, repeated measurements can improve the signal to noise ratio at the expense of an increased measurement time. This proof-of-principle experiment demonstrates the potential of utilizing the proposed time-domain MRX for *in-vitro* diagnostics.

## Discussion

Unlike the traditional magnetometry which only measures the magnetic field, spin relaxometry (i.e., electron spin and nuclear spin) measures the temporal magnetic response arising from the unique atomic structure. Early development on magnetic relaxation for biomedical applications focused on nuclear magnetic resonance (NMR) based on nuclear spin relaxation^46^,^47^. With the endeavor of miniaturization of NMR devices^48^,^49^,^50^,^51^, relaxometry-based microchips have drawn attention recently and are moving toward molecular/cellular diagnostics using electron spin relaxation (ESR). However, both NMR and ESR typically require large magnets to generate the polarizing field limiting their miniaturization. With the state-of-the-art semiconductor technologies, MR-based biochips have the merits of low cost, as well as improved compatibility with lab-on-a-chip systems, integrated electromagnets, and complementary metal-oxide-semiconductor (CMOS) that can be further applied in POC settings using MRX. Although MRX has been investigated for two decades, the lack of systematic study of MNP characteristics for temporal measurement has prevented time-domain MRX from being a reliable biosensing technique. Previously, the deadzone time restrained time-domain MRX from surface immunoassays, urging MRX toward frequency-domain measurements with homogeneous assays. While frequency-domain MRX has shown promise for homogeneous assays^18^,^52^, its high dependence on hydrodynamic volume decreases the distinguishability between analytes. Also, homogeneous assays increase the distance between analytes and sensor surface that would remarkably diminish the magnetic signal 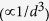. Due to the above challenges in MRX, there is an urgent need to re-innovate time-domain MRX in unprocessed samples without the loss of magnetic sensitivity. Some state-of-the-art works substantially improved the temporal limit of MRX by using Hall-effect sensors and inductive microchips with a high sampling-rate analog to digital converter to capture the dynamic response^20^,^21^,^53^,^54^,^55^. Yet, the realization on bioassay using time-domain GMR MRX hasn’t been reported to date.

In this work, we successfully demonstrated a time-domain MRX for biotin-streptavidin assay using GMR biosensors to investigate the temporal relaxation of commercial MNPs. The experimental investigation was designed on a theoretical basis with the investigation of Néel relaxation of dry MNPs which is prohibited from Brownian relaxation, coverage correlation that demonstrated unequal contribution of signal from stripes and trenches, extraction of characteristic time of different MNPs that proved the feasibility of distinguishing various MNPs in an assay, and the first realization using GMR on time-domain bio-MRX. In summary, the systematic investigation of our work on time-domain MRX enables us to perform bio-MRX with GMR biosensors.

## Methods

### GMR Sensor Chip and Magnetic Nanoparticles

GMR sensor chips were purchased from MagArray. Each GMR chip has 80 individually addressable sensors arranged in 8 × 10 matrix with a nominal resistance (*R*_0_) of 1729 Ω and a magnetoresistance ratio of 11.5%. The sensors do have hysteresis and anisotropy; however, this does not affect the proposed MRX measurement technique since the field is always swept along the same path and the resistance differential is measured. The magnetic particles used in all experiments were coated with streptavidin and purchased from Ocean NanoTech (catalog #: SHS-30-01), Micromod Partikeltechnologie GmbH (Nanomag^®^-D 130 nm, catalog #: 09-19-132), and Thermofisher Scientific (Dynabeads^®^ MyOne™ Streptavidin T1, catalog #: 65601).

### Magnetic Simulation, Modelling, and Fitting

To calculate the MNPs’ average field on a GMR sensor, we adopted Stoner–Wohlfarth (SW) model for magnetic modelling. Assuming MNPs are Langevin spheres in the field regime (2–100 Oe), MNPs have a linear superparamagnetic response and give rise to a dipole field. The volume susceptibilities of SHS-30, Nanomag-D 130 nm, and MyOne at room temperature are 3.60, 4.44, and 1.38 (SI unit, dimensionless), respectively^41^,^42^,^43^. Here, we consider only the spatially averaged magnetic field ***H***_***s***_ on the sensor from MNPs being magnetized by the applied field ***H***_***A***_. Thus, the average field of a single MNP in the free layer 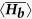 is:


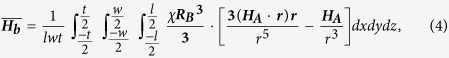


where *l* is the sensor length, *w* is the sensor width, and *t* is the free layer thickness *χ* is the dimensionless magnetic susceptibility, *R*_*B*_ is the MNP radius, *H*_*A*_ is the applied magnetic field, *r* is the distance between MNP and the center point of free layer, *x* and *y* are the in-plane axes, and *z* is the out-of-plane axis. Consequently, we neglect the component along long-axis (***x***, as Fig. 1) of the sensor due to insensitivity of the long-axis field, and only consider the average field along the short-axis (***y***)


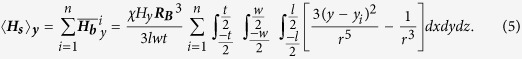


To extract the characteristic time (*t*_*c*_), a MATLAB script was written that incorporates these equations, critical volume approximation^30^, and the signal transduction to calculate the corresponding resistance change as


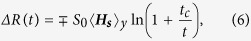


where *S*_0_ is the sensor sensitivity (Ω/Oe), *t* is the time after turning off the field, and *t*_*c*_ is the characteristic time.

### Measurement Setup

**Figure 8:**
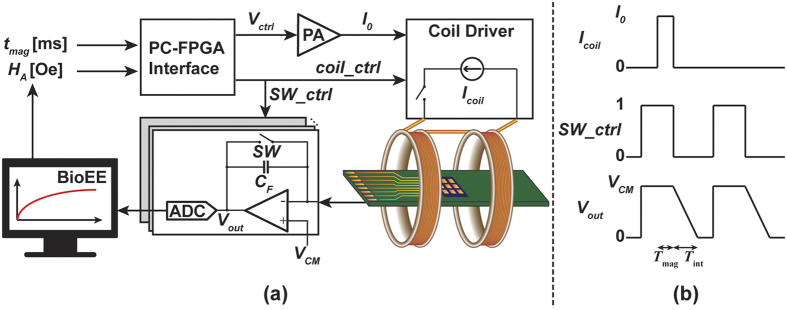
Simplified schematic of measurement system.

The measurement setup consisted of a computer running MATLAB, a field programmable gate array (FPGA, Opal Kelly XEM6310) to control the timing, a power amplifier (PA, Kepco BOP 36–12 ML), a custom designed coil driver and Helmholtz electromagnet, and custom designed readout electronics (Fig. 8). The computer can digitally adjust both the magnetic field and magnetization time through the FPGA. The current from the sensors was integrated and then digitized using a National Instruments data acquisition card (NI PCIe-6351). To remove DC offset, temperature drift, 1/*f* and other correlated noises, and circuit non-linearity, a correlated double sampling (CDS) technique is used where the sensor is sampled two different times: once with the magnetic field and once without the magnetic field. The CDS technique eliminates the need for magnetic shielding, which is extensively used to minimize magnetic noise, circuit non-linearity, and hysteresis^29^. The extracted signal (Δ*V*_out_) can be written as:


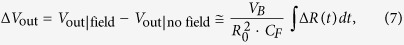


where *V*_*B*_ is the bias voltage (0.5 V), *C*_*F*_ is the integration capacitor, and Δ*R* is the magnetoresistance signal due to the MNP. Multiple measurements were averaged to reduce the white noise and further improve the signal-to-noise ratio (SNR).

### MNP Handling and Coverage Analysis

MNPs were washed with DI water before using, the resulting elimination of salt concentration improved the accuracy of coverage analysis. The MNP coverages were analyzed by MRX signals and SEM images, respectively. A FFT bandpass filter was applied during image processing to increase the contrast between MNPs and sensor substrate through software ImageJ. The MNP concentration was increased to modulate the surface coverage (Table 1).

### Bioassay

The GMR sensors were functionalized with 99% (3-Aminopropyl)triethoxysilane (APTES, catalog #440140, Sigma Aldrich) for 1 hour at 37 °C, followed by Biotin (EZ-Link™ NHS-PEG12-Biotin, catalog #21312, ThermoFisher Scientific) incubated for 1 hour at 37 °C, and then coated with 2% BSA (Blocker™ BSA (10×) in PBS, catalog #37525, ThermoFisher Scientific) for 30 min at room temperature. The reference sensors were covered with epoxy and part of sensors had only BSA without biotin as negative controls. The measurements were conducted with magnetometry to ensure the efficacy of MNPs binding via biotin-streptavidin interaction, followed by 1× PBS washing 3 times to remove unbound MNPs, and then performed MRX to detect the MNPs’ relaxation signal via specific binding.

## Additional Information

**How to cite this article**: Huang, C.-C. *et al*. Giant Magnetoresistive Biosensors for Time-Domain Magnetorelaxometry: A Theoretical Investigation and Progress Toward an Immunoassay. *Sci. Rep.* **7**, 45493; doi: 10.1038/srep45493 (2017).

**Publisher's note:** Springer Nature remains neutral with regard to jurisdictional claims in published maps and institutional affiliations.

## Figures and Tables

**Table 1:** Summary of MNP concentrations.

Cmpany	MNPs	Original Concentration	Concentrated Ratio	Final Concentration
Ocean NanoTechnology	SHS-30	33.9 nM	2×	67.8 nM
Micromod	Nanomag-D 130 nm	4.82 nM	5×	24.1 nM
Thermofisher Scientific	MyOne T1	14.1 pM	100×	1.41 nM
